# LRP1 protects against excessive superior mesenteric artery remodeling by modulating angiotensin II–mediated signaling

**DOI:** 10.1172/jci.insight.164751

**Published:** 2023-01-24

**Authors:** Jackie M. Zhang, Dianaly T. Au, Hisashi Sawada, Michael K. Franklin, Jessica J. Moorleghen, Deborah A. Howatt, Pengjun Wang, Brittany O. Aicher, Brian Hampton, Mary Migliorini, Fenge Ni, Adam E. Mullick, Mashhood M. Wani, Areck A. Ucuzian, Hong S. Lu, Selen C. Muratoglu, Alan Daugherty, Dudley K. Strickland

**Affiliations:** 1 Center for Vascular and Inflammatory Diseases and; 2 Department of Surgery, University of Maryland School of Medicine, Baltimore, Maryland, USA.; 3 Saha Cardiovascular Research Center and Saha Aortic Center and; 4 Department of Physiology, University of Kentucky, Lexington, Kentucky, USA.; 5 Ionis Pharmaceuticals, Carlsbad, California, USA.; 6 Vascular Services, Baltimore Veterans Affairs Medical Center, Baltimore, Maryland, USA.; 7 Department of Physiology, University of Maryland School of Medicine, Baltimore, Maryland, USA.

**Keywords:** Vascular Biology, Cardiovascular disease

## Abstract

Vascular smooth muscle cells (vSMCs) exert a critical role in sensing and maintaining vascular integrity. These cells abundantly express the low-density lipoprotein receptor–related protein 1 (LRP1), a large endocytic signaling receptor that recognizes numerous ligands, including apolipoprotein E–rich lipoproteins, proteases, and protease-inhibitor complexes. We observed the spontaneous formation of aneurysms in the superior mesenteric artery (SMA) of both male and female mice in which LRP1 was genetically deleted in vSMCs (smLRP1^–/–^ mice). Quantitative proteomics revealed elevated abundance of several proteins in smLRP1^–/–^ mice that are known to be induced by angiotensin II–mediated (AngII-mediated) signaling, suggesting that this pathway was dysregulated. Administration of losartan, an AngII type I receptor antagonist, or an angiotensinogen antisense oligonucleotide to reduce plasma angiotensinogen concentrations restored the normal SMA phenotype in smLRP1^–/–^ mice and prevented aneurysm formation. Additionally, using a vascular injury model, we noted excessive vascular remodeling and neointima formation in smLRP1^–/–^ mice that was restored by losartan administration. Together, these findings reveal that LRP1 regulates vascular integrity and remodeling of the SMA by attenuating excessive AngII-mediated signaling.

## Graphical abstract

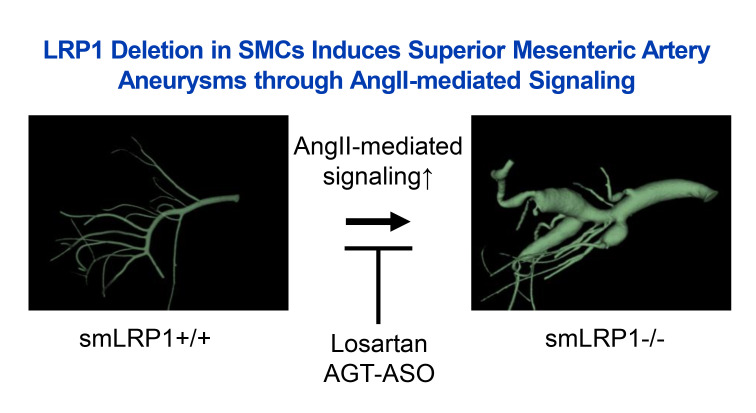

## Introduction

Maintaining vascular integrity is essential for normal physiological function. Loss of integrity leads to formation of aortic aneurysms, which dilate abnormally and may eventually rupture, resulting in life-threatening events. Thoracic aortic aneurysms and dissections typically afflict the young and often result from underlying specific gene mutations (1, 2). In contrast, abdominal aortic aneurysms classically affect older men with significant comorbidities. To date, no single genetic determinant has been identified that is sufficient to cause abdominal aortic aneurysms. Aneurysms are defined clinically as a permanent focal dilation and can occur in vessels outside of the aorta, such as the splanchnic arteries. This arterial bed includes the splenic, celiac, hepatic, superior mesenteric, and inferior mesenteric arteries. Splanchnic artery aneurysms occur with an estimated incidence of 0.1%–2% of the adult population (3), and currently, very little is known about the mechanism associated with their development. It is likely that aneurysms in these vessels occur via similar mechanisms that have been associated with aortic aneurysms. Recent studies in mice have identified a critical role for the low-density lipoprotein receptor–related protein 1 (LRP1) in protecting against aortic aneurysms (4–8).

LRP1 is a large endocytic signaling receptor that contributes to vascular development (9), exerts a role in lipoprotein metabolism (10, 11), regulates protease concentrations (5, 12, 13), regulates inflammation (14), and attenuates the progression of atherosclerosis and aneurysm formation (4–8). Genetic studies in humans have revealed that the LRP1 gene is a susceptibility locus for aortic aneurysms and dissections (15–21). The mechanisms by which LRP1 protects the vasculature are not fully understood but may involve regulating platelet-derived growth factor receptor (PDGFR) activation (4, 22–24), modulating transforming growth factor-β (TGF-β) and connective tissue growth factor (CTGF) signaling (5, 7, 25–28), and regulating protease concentrations in the vessel wall (5). Additionally, LRP1 regulates vascular smooth muscle cell (vSMC) contraction (6) and proliferation (6, 29). In mice, genetic deletion of LRP1 in vSMCs (smLRP1^–/–^) results in development of spontaneous thoracic aneurysms, as well as abnormal medial wall thickening and degradation and fragmentation of the elastic laminae (5). Interestingly, chronic infusion of angiotensin II (AngII) into smLRP1^–/–^ mice results in pronounced superior mesenteric artery (SMA) medial thickening, neointimal formation, elastic fragmentation, a dramatically exacerbated dilatation, and a high rate of rupture (30).

AngII-mediated signaling has been studied frequently in the cardiovascular field as the renin-angiotensin system exerts a key role in regulating systemic vascular resistance and maintaining arterial structure (31, 32). Angiotensinogen (AGT), a member of the serine protease inhibitor family, is predominantly secreted from the liver (33, 34) and cleaved by renin to produce angiotensin I, which is subsequently cleaved in a reaction catalyzed by angiotensin-converting enzyme to produce AngII. AngII signals via 2 receptors, AngII receptor type 1 (AGTR1) and AngII receptor type 2 (AGTR2), and increases neointimal hyperplasia development in response to vascular injury (35–38). Moreover, the renin-angiotensin system also contributes to development and progression of aortic aneurysms and dissections (39–42). Experimentally, AngII infusion into mice is used widely as a model for aortic aneurysms and dissections (43). AngII-mediated signaling also upregulates LRP1 in vSMCs isolated from rat aorta (44) and upregulates several LRP1 ligands, including plasminogen activator inhibitor 1 (45), protease nexin 2 (serpine2) (46), TGF-β (47), CTGF (48), and matrix metalloproteinase 2 (MMP-2) (49).

We noted spontaneous and fully penetrant formation of SMA aneurysms in both male and female smLRP1^–/–^ mice. The objective of the current investigation was to define molecular mechanisms by which LRP1 protects against aneurysm formation in this vessel bed. We also used a vascular injury model dependent upon AngII-mediated signaling to study the contribution of LRP1 in this process. Our results revealed that LRP1 maintains vascular wall integrity and regulates vascular remodeling in these arteries by attenuating AngII-mediated signaling.

## Results

### Spontaneous dilation and remodeling of the SMA in smLRP1^–/–^ mice.

**Figure 1:**
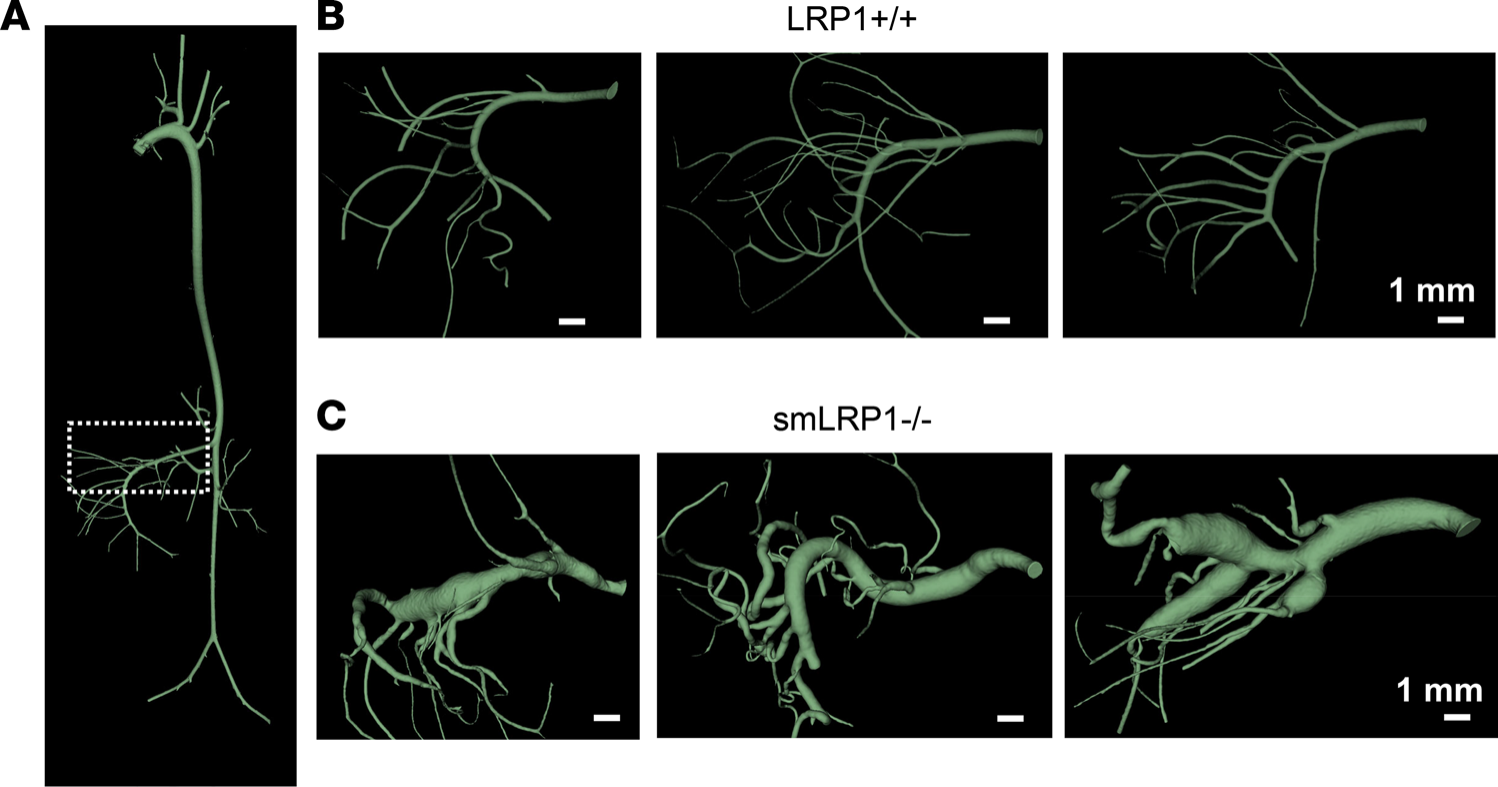
Reconstruction in 3D of micro-CT images reveals substantial SMA pathology in smLRP1^–/–^ mice. CT scans from Microfil-infused mice were reconstructed using the 3D Slicer program. (**A**) Image of the entire aorta from an LRP1^+/+^ mouse. Boxed area identifies the SMA. (**B** and **C**) Reconstruction in 3D of the SMAs from 40-week-old LRP1^+/+^ (**B**) and smLRP1^–/–^ mice (**C**). (Scale bar = 1 mm.)

**Figure 2:**
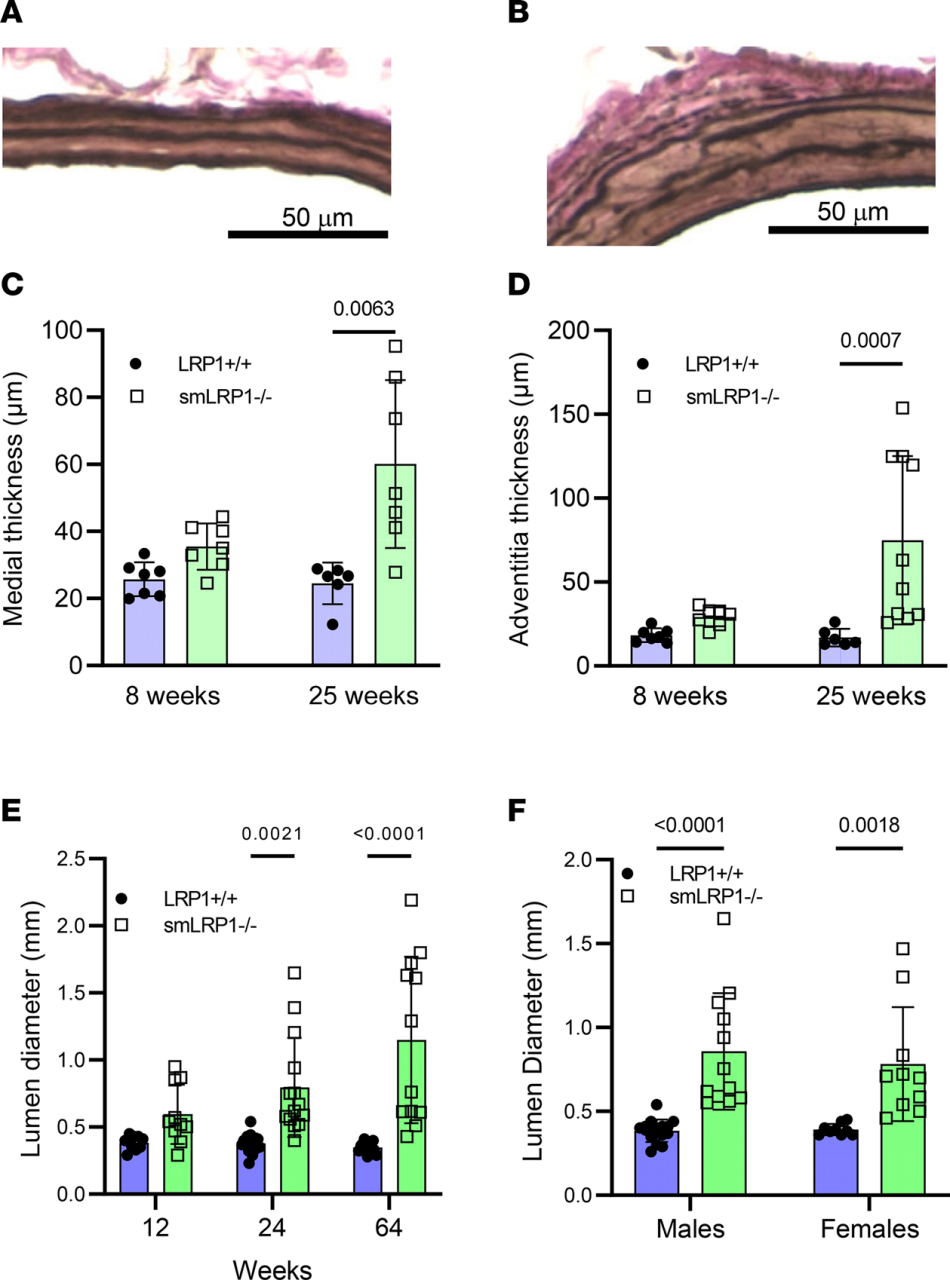
Remodeling of the SMAs in smLRP1^–/–^ mice. (**A** and **B**) Representative elastin van Gieson staining of SMA sections from 16-week-old LRP1^+/+^ (**A**) or smLRP1^–/–^ (**B**) mice. (**C** and **D**) Morphometric measurements of SMA medial thickness (**C**) and adventitial thickness (**D**) in 8-week-old and 25-week-old mice. (**E**) Lumen diameter measured from micro-CT measurements for SMA in 12-, 24-, and 64-week-old mice. (**F**) Lumen diameter determined from micro-CT measurements for SMA in 16-week-old male and female mice. (**C**, **D**, and **F**, Kruskal-Wallis nonparametric test, Dunn’s multiple-comparison test; **E**, 2-way ANOVA, Tukey’s post hoc test comparing LRP1^+/+^ and smLRP1^–/–^ at each age.)

Our prior studies have confirmed effective deletion of LRP1 from smooth muscle cells (SMCs) in smLRP1^–/–^ mice (5, 30). Micro-CT imaging of vasculature of LRP1^+/+^ (Figure 1, A and B) and smLRP1^–/–^ mice (Figure 1C) revealed extensive dilatation of the SMAs in smLRP1^–/–^ mice. Histological analyses of SMAs from LRP1^+/+^ (Figure 2A) and smLRP1^–/–^ mice (Figure 2B) at 16 weeks of age revealed profound degradation of elastic laminae in smLRP1^–/–^ mice. Morphometric measurements confirmed that remarkable thickening of the media (Figure 2C) and adventitia (Figure 2D) occurred as the mice aged. There was no notable neointima formation in the SMAs of smLRP1^–/–^ mice. Ex vivo measurements using micro-CT imaging of the SMA lumen diameter revealed that the SMA lumen diameter increased significantly in smLRP1^–/–^ mice at 24 and 64 weeks of age (Figure 2E). This was observed in both male and female smLRP1^–/–^ mice (Figure 2F). There was no noticeable difference in the SMA lumen diameter between sexes regardless of genotype (Figure 2F).

**Figure 3:**
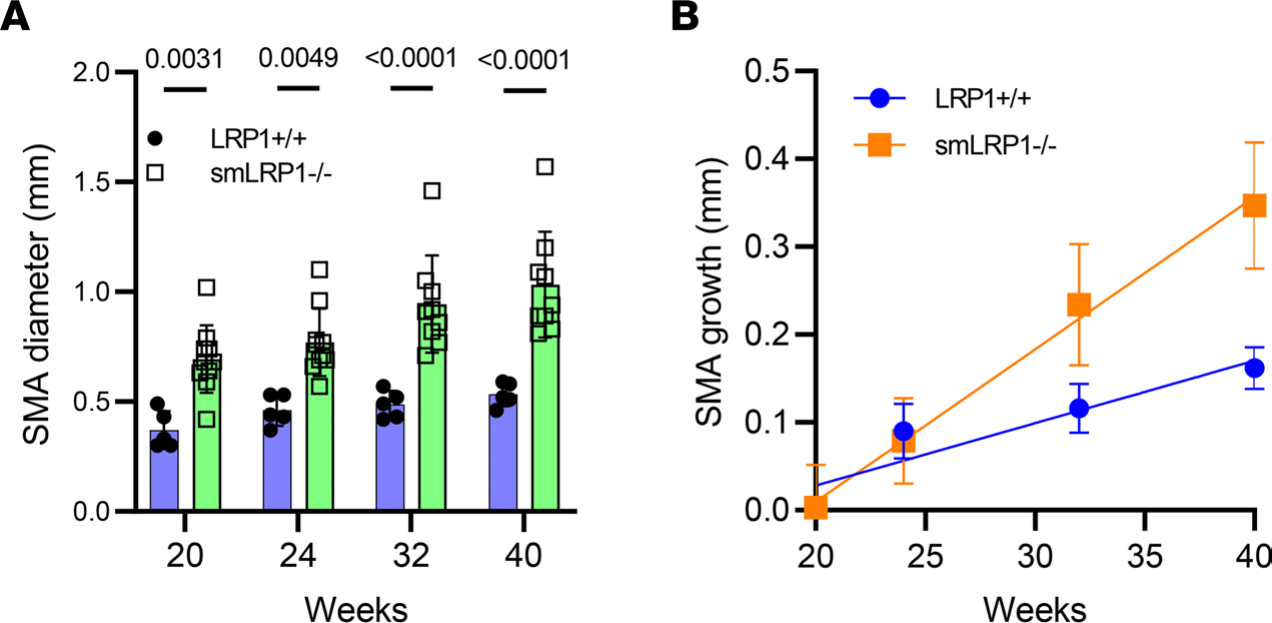
Ultrasonography reveals that the SMA diameter expands in smLRP1^–/–^ mice at an exceptional rate when compared with WT mice. (**A**) Ultrasonography was performed on mice at 20, 24, 32, and 40 weeks of age. Maximal lumen diameters were measured on the captured images (2-way ANOVA with Tukey’s post hoc test comparing LRP1^+/+^ and smLRP1^–/–^ mice at each age). (**B**) Rates of SMA growth using the 20-week measurements as baseline (mean ± SEM; *n* = 5 WT; *n* = 10 smLRP1^–/–^). (Data were fit to a straight line using linear regression analysis available in Prism 9.0 by GraphPad Software. Slopes were determined to be different, *P* = 0.01.)

Upon ultrasound measurements of maximal lumen diameters of the SMA, at 20 weeks of age and beyond, enhanced vessel dilatation of the SMA upon was observed in smLRP1^–/–^ when compared with LRP1^+/+^ mice (Figure 3A). The rate of SMA dilatation, when measured at 20 weeks (as baseline) over a subsequent 20-week period, revealed a 2-fold increase in the rate in smLRP1^–/–^ when compared with LRP1^+/+^ mice (Figure 3B).

### Global proteomic analyses reveal activation of the AngII and TGF-β signaling pathways in SMAs of smLRP1^–/–^ mice.

**Figure 4:**
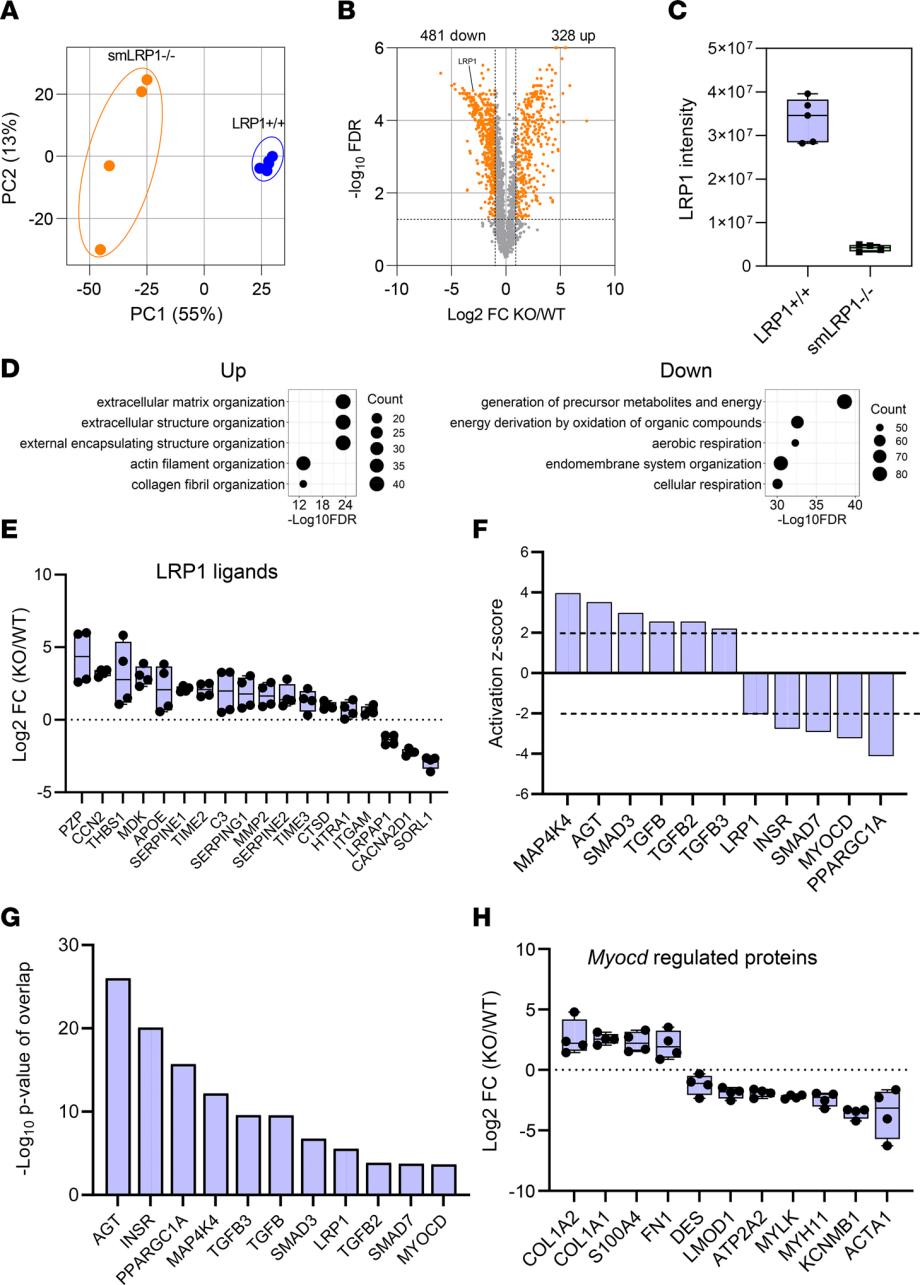
Proteomic analyses reveal activation of AngII signaling pathways in SMAs of smLRP1^–/–^ mice. (**A**) Principal component analysis of the LRP1^+/+^ (*n* = 5) versus smLRP1^–/–^ (KO) (*n* = 4) samples from 14-week-old mice. (**B**) Volcano plot showing –log_10_ FDR (*y* axis) versus log_2_ fold-change (FC) for each protein (orange, FDR < 0.01, FC > |2|; gray, FDR > 0.01). (**C**) Intensity levels for LRP1 in LRP1^+/+^ and smLRP1^–/–^ mice. (**D**) Gene ontology enrichment analysis for upregulated (*left panel*) or downregulated (*right panel*) pathways. (**E**) Log_2_ fold-change for selected LRP1 ligands as determined by mass spectral intensities. (**F**) Activation *z* scores for selected pathways and (**G**) *P* values of overlap for pathways identified in IPA software. (**H**) Fold-changes in myocardin-regulated proteins in smLRP1^–/–^ mice relative to LRP1^+/+^ as quantified by mass spectrometry.

To identify potential mechanisms by which LRP1 regulates vascular remodeling, we used quantitative proteomic analysis to characterize the molecular signatures that may be integral in contributing to the phenotype observed in the SMAs of smLRP1^–/–^ mice. Principal component analysis revealed distinct clusters for LRP1^+/+^ versus smLRP1^–/–^ proteomes (Figure 4A). Using a fold-change value of 2 and FDR < 0.01, proteomic analyses identified 2,465 total proteins, of which 809 were significantly altered in smLRP1^–/–^ SMAs when compared with LRP1^+/+^ mice (Figure 4B). Mass spectrometry data supported a greater than 8-fold decrease in LRP1 abundance in the SMAs of smLRP1^–/–^ mice (Figure 4C). Gene ontology enrichment analysis of upregulated proteins revealed major clusters in categories associated with extracellular matrix organization, actin filament organization, and collagen fibril organization (Figure 4D, *left panel*), while gene ontology analysis of downregulated proteins revealed major clusters in energy metabolism and membrane organization (Figure 4D, *right panel*). The intensity level of LRP1 ligands derived from the mass spectral analysis demonstrated that many LRP1 ligands were elevated in SMA tissue of smLRP1^–/–^ mice relative to LRP1^+/+^ mice (Figure 4E). We also noted from the mass spectral analysis that integrin subunits and proteins involved in integrin function were downregulated in the SMAs of smLRP1^–/–^ mice (Supplemental Table 1; supplemental material available online with this article; https://doi.org/10.1172/jci.insight.164751DS1). These include the α subunit of several integrins as well as talin 1 and kindlin-2, both of which interact with integrin cytoplasmic tails, leading to integrin activation (50). Together, these observations imply that a major defect in the SMAs of smLRP1^–/–^ mice involves integrin/matrix interactions, which are critical for normal vSMC function (51).

Casual analysis of the proteomic data (52) in Ingenuity Pathway Analysis (IPA) software revealed a high probability for the activation of a number of pathways known to influence vascular remodeling (Figure 4F) with a high degree of significance (Figure 4G). These pathways included the AngII-mediated (activation *z* score of 3.1; *P* value of overlap = 7.9 × 10^–17^) and TGF-β1 signaling pathways (*z* score = 2.0; *P* value of overlap = 6.3 × 10^–23^), both of which are associated with vascular remodeling (43, 53). Interestingly, these data also predicted that the SMAD7 pathway was inhibited (*z* score = –3.5; *P* value of overlap = 1.4 × 10^–5^). SMAD7 functions as an inhibitor of TGF-β signaling by associating with the E3 ubiquitin ligase SMURF2, which triggers degradation of the TGF-β receptor type 1 (54, 55).

### Global proteomic analyses predict inhibition of transcriptional programing that regulates SMC differentiation.

Of additional interest, our proteomic data predicted that myocardin-mediated signaling was inhibited in the SMAs of smLRP1^–/–^ mice (*z* score = –2.8; *P* value of overlap = 8.9 × 10^–4^). Myocardin is a nuclear protein expressed in SMCs that plays a crucial role in differentiation of SMCs (56). The intensity levels of proteins derived from the mass spectral analysis that are regulated by myocardin are shown in Figure 4H and include several proteins, such as Myh11, which is associated with a mature contractile SMC phenotype. Interestingly, missense mutations in the MYH11 gene are associated with thoracic aortic aneurysms (57).

### Global proteomic analyses predict deregulation of proteinases and proteinase inhibitors in the SMA.

Proteomic data comparing the SMAs from smLRP1^–/–^ mice with WT mice revealed that several proteases and protease inhibitors were dysregulated (Supplemental Figure 1, A and B). These included members of the ADAMTS family, MMPs, coagulation proteases, as well as members of the cathepsin family of proteinases. Interestingly, SERPINC1 (antithrombin III) was decreased in smLRP1^–/–^ mice, suggesting excessive thrombosis in smLRP1^–/–^ mice.

### Inhibition of AngII-mediated signaling restores the SMA phenotype.

**Figure 5:**
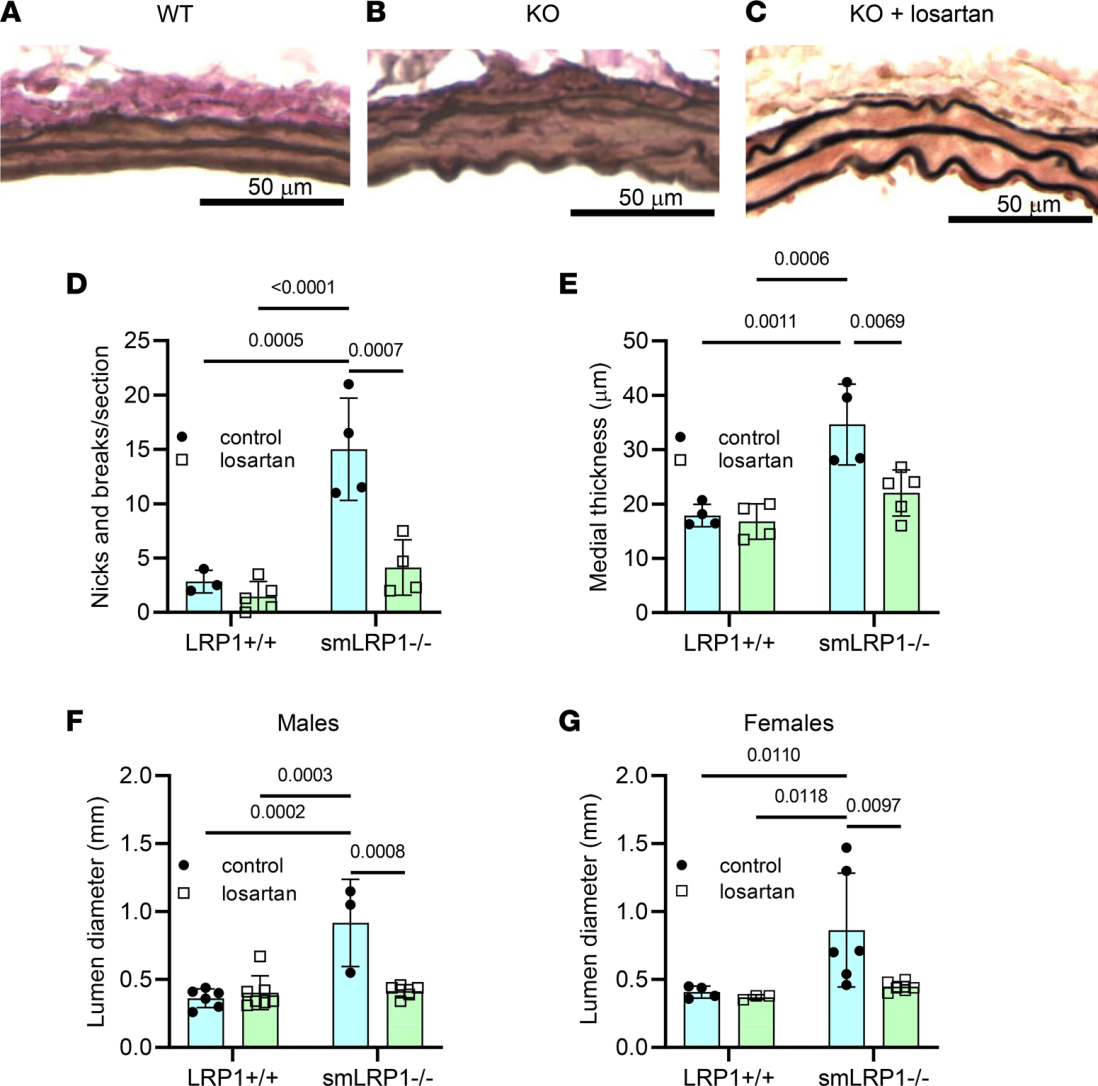
Losartan administration restores the arterial phenotype of smLRP1^–/–^ mice. After weaning at 3–4 weeks of age, mice were provided with or without losartan (0.6 g/L drinking water) and kept on the drug for 12 weeks before analysis. (**A**–**C**) Elastic van Gieson (EVG) staining of sections from LRP1^+/+^ SMAs (**A**) or smLRP1^–/–^ (**B**) mice or (**C**) smLRP1^–/–^ mice administered with losartan. smLRP1^–/–^ mice receiving losartan had fewer breaks in the elastic laminae (**D**) and reduced medial thickening (**E**) than those observed in smLRP1^–/–^ mice. Micro-CT measurements of lumen diameter for male (**F**) and female (**G**) LRP1^+/+^ or smLRP1^–/–^ mice with or without losartan treatments (2-way ANOVA, Tukey’s multiple-comparison test).

Since our proteomic data were consistent with AngII-mediated signaling being activated in smLRP1^–/–^ mice, we designed experiments to further investigate the role of the AngII signaling pathway on SMA dilatation. To accomplish this, we elected to pharmacologically block this pathway by administering losartan, an AGTR1 antagonist that selectively blocks the binding of AngII to AGTR1. During administration, we monitored systolic and diastolic blood pressure, which was reduced in both LRP1^+/+^ and smLRP1^–/–^ mice comparably (Supplemental Figure 2). Histological analysis, as well as morphometric measurements, revealed that AGTR1 blockade with losartan reduced elastic laminae degradation (Figure 5, A–D) and medial thickening in these mice at 16 weeks of age (Figure 5E). In addition, there was a significant decrease in the SMA lumen diameter as measured from micro-CT imaging in both male (Figure 5F) and female (Figure 5G) smLRP1^–/–^ mice. These data revealed that losartan prevented formation of SMA aneurysms in smLRP1^–/–^ mice and support results obtained from our proteomic data, revealing that AngII signaling exerts a critical role in SMA pathology in smLRP1^–/–^ mice.

### Reduction in plasma AGT concentrations restores the SMA phenotype.

**Figure 6:**
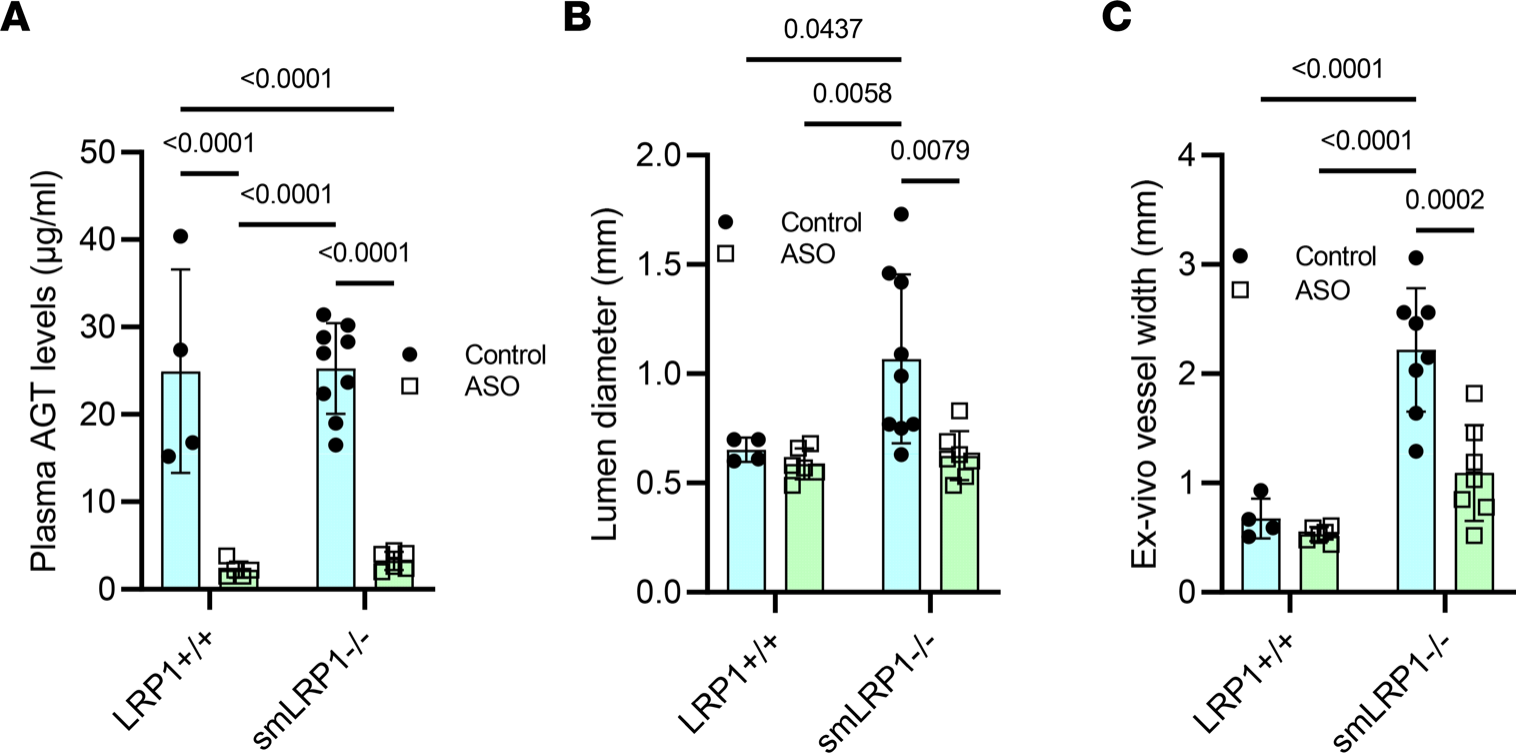
AGT ASO administration restores the arterial phenotype of smLRP1^–/–^ mice. Six-week-old mice were injected subcutaneously with AGT ASO on days 1 and 4 and then weekly for 11 weeks. At 18 weeks of age, mice were sacrificed. (**A**) Plasma AGT concentrations were quantified by ELISA; (**B**) lumen diameters of SMAs were quantified by ultrasonography; and (**C**) ex vivo widths of SMAs from LRP1^+/+^ and smLRP1^–/–^ mice were measured (2-way ANOVA, Tukey’s multiple-comparison test).

To assess the contribution of plasma-derived AGT on SMA remodeling in smLRP1^–/–^ mice, we employed AGT antisense oligonucleotide (ASO) to reduce plasma AGT concentrations. This approach significantly reduced plasma AGT concentrations in both LRP1^+/+^ and smLRP1^–/–^ mice (Figure 6A). Twelve weeks following ASO administration, ultrasound measurements confirmed that the lumen diameter of the SMAs in smLRP1^–/–^ mice was indistinguishable from those of LRP1^+/+^ mice (Figure 6B). Further, ex vivo measurements of SMA vessel width (Figure 6C and see Supplemental Figure 3 for examples) also revealed no difference in AGT ASO–administered smLRP1^–/–^ and LRP1^+/+^ mice (Figure 6C). These results support the contribution of AGT-mediated signaling to the SMA phenotype.

AGT has been reported to bind directly to LRP1 (58), and thus we considered the possibility that LRP1 expressed in vSMCs might regulate plasma AGT concentrations by binding this ligand and mediating its internalization and degradation. An ELISA supported equal concentrations of both AGT and renin in plasma of LRP1^+/+^ and smLRP1^–/–^ mice (Supplemental Figure 4, A and B), indicating that LRP1 deficiency in SMCs did not affect AGT concentrations in plasma.

### LRP1 expression attenuates vascular remodeling upon injury by regulating AngII-mediated signaling.

**Figure 7:**
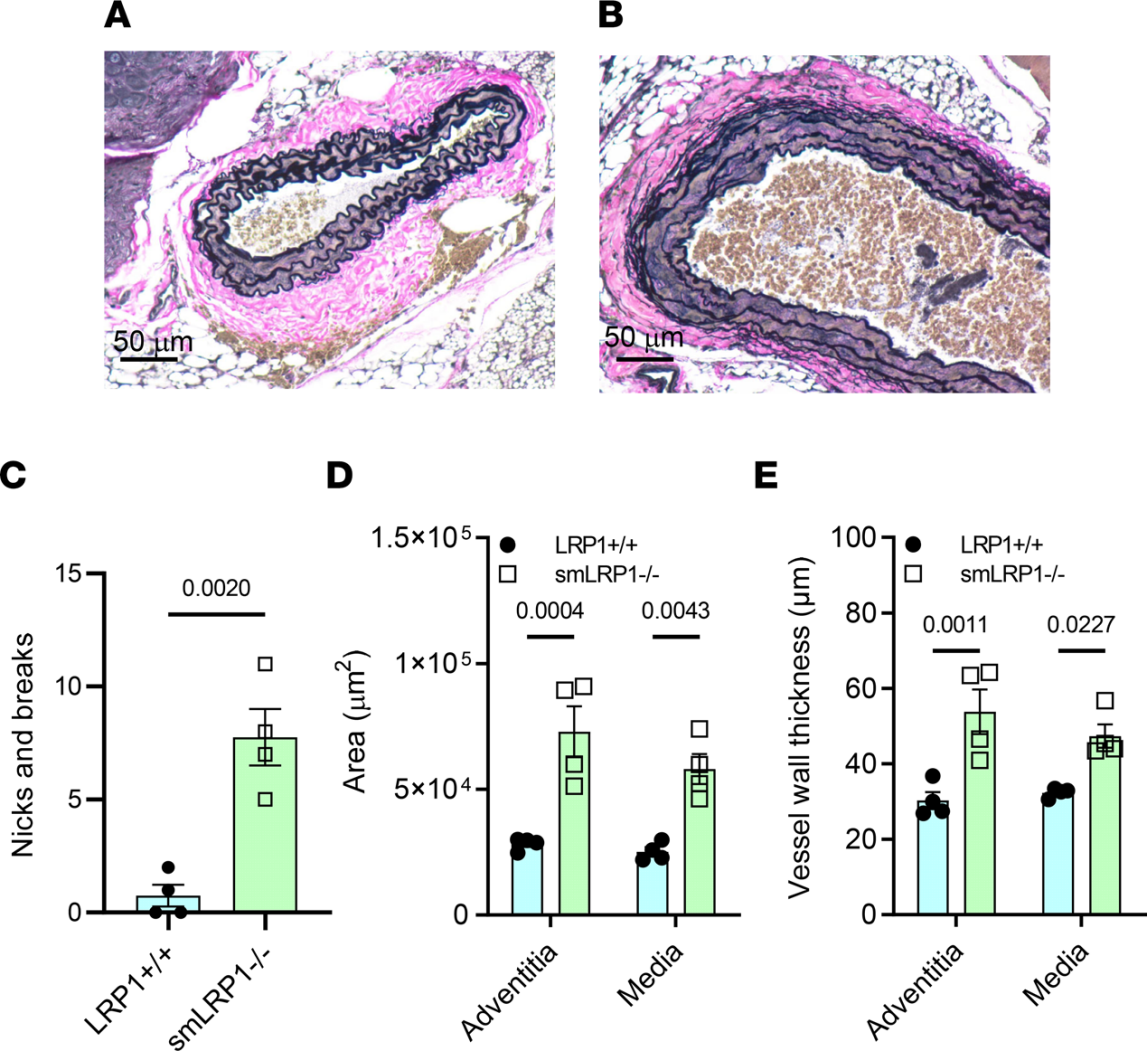
Remodeling of the carotid artery in smLRP1^–/–^ mice. (**A** and **B**) EVG staining of carotid arteries from 1-year-old LRP1^+/+^ (**A**) or smLRP1^–/–^ mice (**B**). (**C**) Breaks in elastic laminae are shown for LRP1^+/+^ and smLRP1^–/–^ mice (Student’s unpaired 2-tailed *t* test). Area (**D**) and thickness (**E**) of the media and adventitia were measured (2-way ANOVA, Tukey’s multiple-comparison test comparing LRP1^+/+^ and KO mice).

To further test the hypothesis that LRP1 attenuates AngII-mediated signaling, we used an established vascular injury model (59) that is known to be mediated by AngII-mediated signaling (38). Initially, we examined the carotid arteries of LRP1^+/+^ and smLRP1^–/–^ mice. Like the SMAs, extensive vascular remodeling occurred in the carotid arteries of smLRP1^–/–^ mice, which resulted in extensive degradation of the elastic laminae (Figure 7, A–C), an increase in the total areas of the adventitia and media (Figure 7D), and medial and adventitial thickening in smLRP1^–/–^ mice (Figure 7E). These data support that LRP1 deficiency also regulated the integrity of this vascular bed.

**Figure 8:**
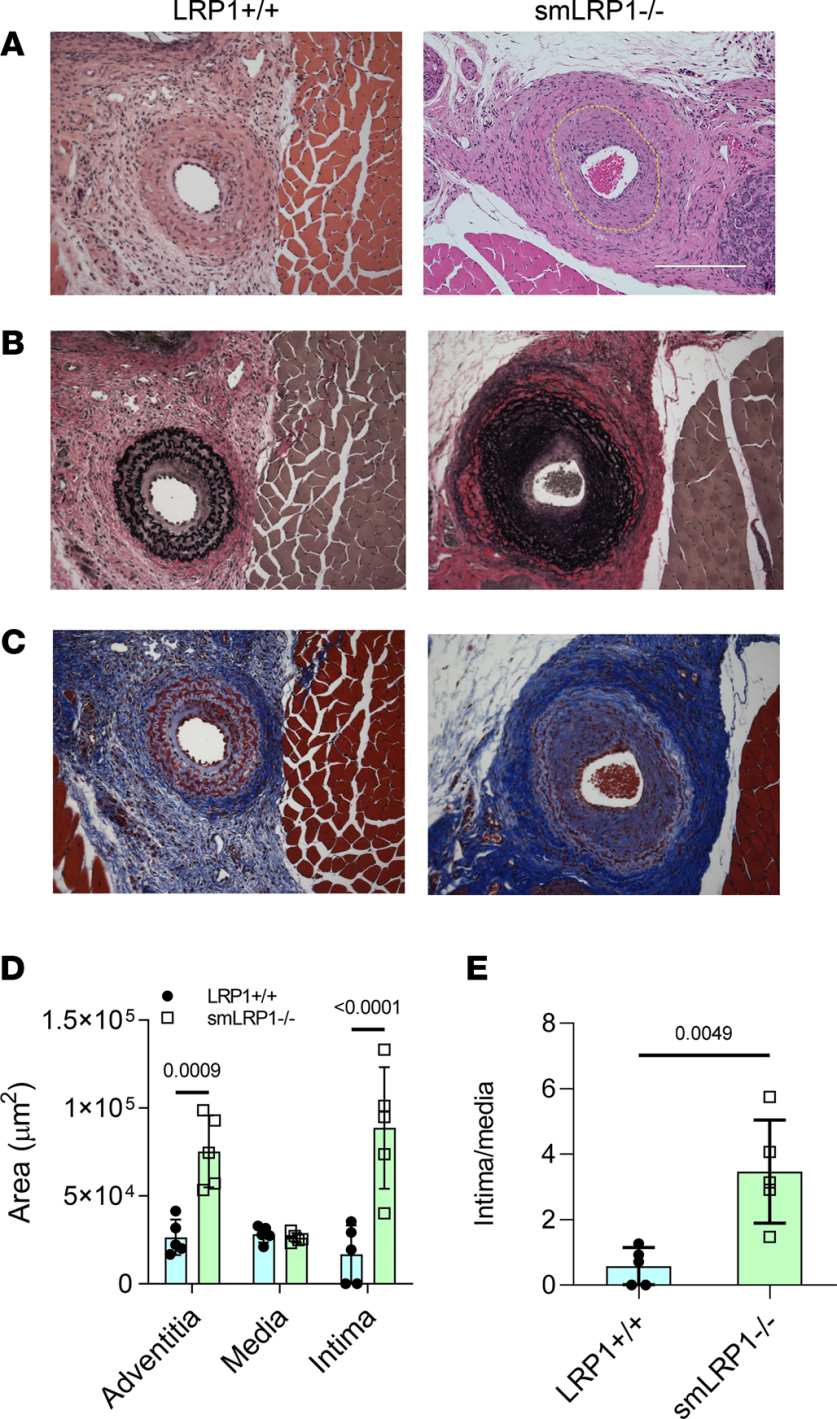
LRP1 protects against vascular remodeling induced by injury. Adult mice (12–16 weeks of age) were subjected to ligation of the left common carotid artery. Four weeks postsurgery, animals were euthanized, and whole-neck serial cross sections of 5 μm thickness were sliced starting from the carotid bifurcation to the area inferior to the lesion apex. (**A**) The apex of the lesion area was identified by analyzing serial sections at 100 μm intervals by H&E, (**B**) EVG, and (**C**) Masson’s trichrome staining. (Scale bar = 200 μm.) Morphometric measurements (**D** and **E**) were performed using EVOS FL Auto Imaging System software (Invitrogen, Thermo Fisher Scientific) (unpaired 2-tailed Student’s *t* test).

In the vascular injury model, LRP1^+/+^ and smLRP1^–/–^ mice at 12–16 weeks of age were subjected to ligation of the left common carotid artery. Four weeks following surgery, mice were euthanized, and the whole neck and head were dissected from each animal. Histological analysis of whole-neck sections by H&E staining (Figure 8A), EVG staining (Figure 8B), and Masson’s trichrome staining (Figure 8C) showed extensive vascular remodeling in smLRP1^–/–^ mice with significant neointima formation (*P* < 0.0001, Figure 8D) compared with LRP1^+/+^ mice. Morphometric measurements verified significant increases in the adventitia and neointima in the carotid arteries of smLRP1^–/–^ mice upon injury (Figure 8, D and E).

**Figure 9:**
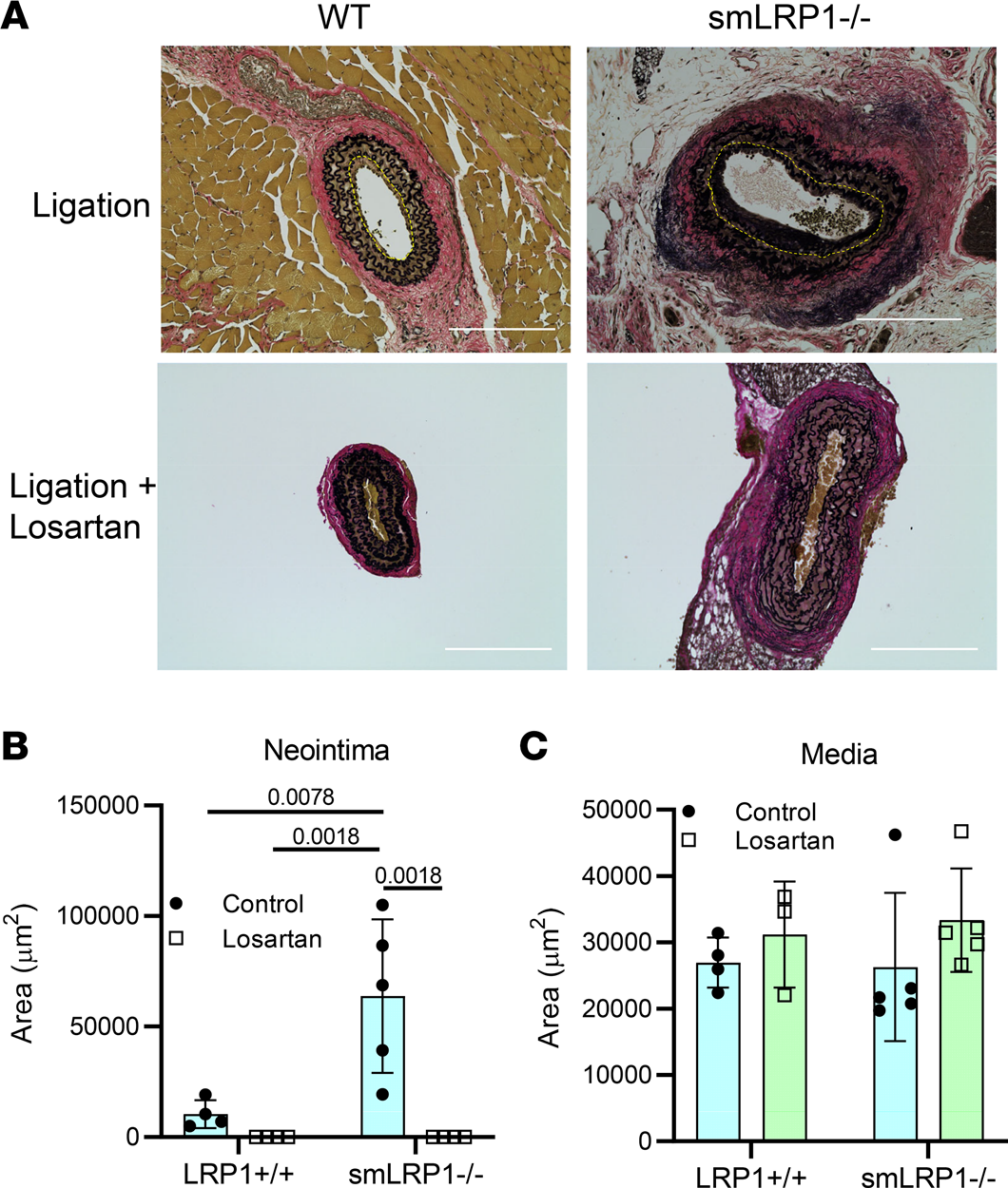
Losartan reduces vascular remodeling induced by injury in smLRP1^–/–^ mice. Adult mice (12–16 weeks of age) were subjected to ligation of the left common carotid artery. Following ligation, mice were provided with or without losartan. Four weeks postsurgery, animals were euthanized, and serial cross sections of 5 μm thickness were sliced starting from the carotid bifurcation to the area inferior to the lesion apex. (**A**) The apex of the lesion area was identified by analyzing serial sections at 100 μm intervals by EVG. (Scale bar = 200 μm.) (**B** and **C**) Morphometric measurements were performed using EVOS FL Auto Imaging System software (Invitrogen, Thermo Fisher Scientific) (2-way ANOVA, Tukey’s multiple-comparison test).

To evaluate the impact of LRP1 deletion on AngII-mediated signaling in this model, LRP1^+/+^ and smLRP1^–/–^ mice were subjected to carotid ligation without or with losartan (0.6 g/L) in their drinking water. Histological analyses of sections stained with EVG (Figure 9A) and morphometric measurements of the vessels demonstrated that losartan administration ablated neointima formation in smLRP1^–/–^ mice (Figure 9, B and C). These results support a major role for LRP1 in regulating vascular remodeling by attenuating excessive AngII-mediated signaling.

## Discussion

We investigated the spontaneous formation of SMA pathology that occurred in both male and female smLRP1^–/–^ mice. The intrinsic characteristics of LRP1-deficient vSMCs resulted in SMA vessel wall architectures that exhibited hallmark characteristics of a damaged vessel wall undergoing vascular remodeling (60, 61). These characteristics include disorganization and fragmentation of elastic fibers, medial thickening due to increased matrix deposition, significant adventitial thickening (Figure 2D), and higher abundance of extracellular matrix–degrading proteases. Quantitative global proteomics revealed that vSMCs in the SMAs of LRP1^–/–^ mice have a phenotype in which contractile genes are downregulated, while extracellular matrix proteins are upregulated. Further, in smLRP1^–/–^ mice, several myocardin-regulated proteins were downregulated. This is of interest, as conditional deletion of the *Mycod* in vSMCs in mice results in arterial aneurysms, dissections, and rupture (56). The findings suggest that LRP1 preserves vascular integrity, at least in part, by promoting myocardin-mediated signaling, which is important for maintaining the contractile function of vSMCs. In summary, our studies reveal that deletion of LRP1 in SMCs results in (a) defective SMC differentiation, (b) defective matrix-SMC interactions via integrins, (c) an injury response associated with upregulation of AngII-targeted and TGF-β–targeted genes, and (d) deregulation of numerous proteinases known to be capable of degrading the matrix.

Upstream regulator analysis (52) of our proteomic results from the SMAs of smLRP1^–/–^ mice revealed dysregulation of the AngII- and TGF-β–mediated signaling pathways. These signaling pathways are of interest, as their excessive activation can result in aneurysm formation (43, 62, 63). To test the hypothesis that dysregulation of AngII-mediated signaling events were causative for the SMA phenotype in smLRP1^–/–^ vessels, we used the AGTR1 antagonist losartan. Clinically, losartan is used commonly to treat hypertension. However, losartan has gained traction as a potential drug to attenuate vascular remodeling in various disease states and is indicated for left ventricular hypertrophy in hypertension patients and nephropathy in type 2 diabetes patients (64, 65). Most recently, losartan has been investigated in long-term clinical trials in patients with Marfan syndrome to improve overall survival by means of preventing aortic dissection and reducing aortic root dilation (66). In addition to lowering blood pressure, losartan antagonizes the TGF-β signaling pathway, presumably through an AngII-based mechanism. In mouse models of Marfan syndrome or Loeys-Dietz syndrome, losartan attenuates vascular remodeling, prevents aortic aneurysms, and improves vessel wall structure (62, 67).

The results of our studies revealed that losartan was highly effective in restoring SMA integrity by reducing lumen diameter, medial thickening, and degradation of the elastic laminae. Losartan administration prevented SMA aneurysm formation in both male and female smLRP1^–/–^ mice. To verify and extend the results obtained by AGTR1 receptor blockade, we also used ASO-mediated AGT-knockdown experiments. Reducing plasma AGT concentrations also restored the phenotype in smLRP1^–/–^ mice. Together, these data provide convincing evidence that a major mechanism by which LRP1 regulates SMA remodeling is via attenuation of AngII-mediated signaling. Previously, Davis et al. (30) revealed that chronic AngII infusion into smLRP1^–/–^ mice resulted in disproportionate SMA pathology and death from mesenteric rupture compared with their LRP1^+/+^ counterparts, which is consistent with our proteomic analysis. However, in their study, they also found that chronic infusion of norepinephrine to promote similar increases in hemodynamic pressure comparable to AngII infusion also produced SMA aneurysms, confounding the relationship between angiotensin and the mechanism(s) by which losartan influences SMA pathology in smLRP1^–/–^ mice. Interestingly, without AngII infusion, the systolic and diastolic blood pressure of 1-year-old smLRP1^–/–^ mice is significantly lower than their LRP1^+/+^ littermates (5), and as shown here, they still develop SMA pathology. Therefore, we conclude that the effects of losartan on SMA pathology seen in smLRP1^–/–^ mice may partially be dependent upon reduction of blood pressure but are also exacerbated by AngII-mediated signaling events that are not solely associated with its elevating hemodynamic effects.

We further tested the potential of LRP1 to modulate AngII-mediated signaling by using a well-characterized model of vascular remodeling (59) known to depend upon AngII-mediated signaling (38). Our results revealed significant neointima formation and adventitial thickening in smLRP1^–/–^ mice when compared with LRP1^+/+^ mice (Figure 7D), supporting that LRP1 protects against injury-induced vascular remodeling. These results concur with those from Basford et al., who used an endothelial denudation model to induce vascular remodeling (29). Importantly, our data revealed that losartan completely blocked the excessive neointima formation noted in the smLRP1^–/–^ mice upon vascular injury. These data provide additional supporting evidence that LRP1 exerts a role in attenuating AngII-mediated signaling events.

Together, these results show that LRP1 exerts a critical role in regulating AngII-mediated signaling events, and in the absence of LRP1, the SMA is spontaneously remodeled in a process that is prevented by AGTR1 blockade or reduction of plasma AGT concentrations. We propose that LRP1 prevents excessive remodeling of the SMA by regulating SMC phenotype and by attenuating AngII-mediated signaling. Our studies raise the possibility that mutations in LRP1 may result in receptor defects that contribute to SMA pathology in human patients. In humans, genome-wide association studies, exome sequencing, and TaqMan single nucleotide polymorphism (SNP) genotyping assays have identified an association of LRP1 SNPs with aortic aneurysms (15–17, 20), aortic dissections (18, 19), and Marfan syndrome (68). Interestingly, aortic, but not plasma, concentrations of soluble forms of LRP1 were significantly lower in patients with abdominal aortic aneurysm (AAA) compared with controls (69). Chan et al. (70) also reported a significant reduction in LRP1 protein abundance in human AAA samples from a Chinese population and have recently demonstrated that translational inhibition by microRNA-205 is responsible for driving the lower abundance of LRP1 (71). Furthermore, lower levels of LRP1 were speculated to result in accumulation of excess MMP-9, a well-documented protease that contributes to degradation of the extracellular matrix proteins, leading to AAA (72). Based on our studies in mice, we hypothesize that rare variants in LRP1 might contribute to SMA pathology in patients.

The process by which LRP1 attenuates AngII-mediated signaling is likely to involve multiple mechanisms as LRP1 is known to regulate the abundance of several important signaling molecules as well as matrix molecules (73) and affects multiple signaling events. Since the blood pressure reductions induced by losartan were not different between LRP1^+/+^ mice and smLRP1^–/–^ littermates, we conclude that LRP1 most likely affected downstream signaling events mediated by AGTR1. Several studies have demonstrated a relationship between AngII and TGF-β signaling in vascular tissue and remodeling (74) as AngII-mediated signaling increases the production of TGF-β (75, 76). Thus, in transgenic mice expressing mutant forms of cardiac troponin T, the interstitial fibrosis that is driven by TGF-β signaling was attenuated with losartan (77). The role of TGF-β in vascular remodeling has been well established (27, 78–83). Additionally, aneurysms in AngII-infused ApoE^–/–^ mice have also been associated with the increased expression of TGF-β in whole-genome expression analysis (84), suggesting a possible synergic effect between TGF-β and AngII signaling. Further, excessive TGF-β signaling was detected in mouse models of Marfan syndrome, and a TGF-β neutralizing antibody, as well as losartan, partially reversed vascular manifestations of Marfan’s syndrome (62). These studies support the notion that LRP1 may affect vascular remodeling, in part, by attenuating TGF-β signaling pathways. This is strengthened by the findings that LRP1 binds to all forms of TGF-β (25, 27) and that LRP1 expressed in macrophages attenuates TGF-β signaling upon vascular injury in mice fed a Western diet (27). Further, liver-specific deletion of LPR1 in mice accelerates liver disease progression in mouse models by increasing sensitivity of profibrotic gene expression to promote steatohepatitis (85).

It is also possible that the LRP1-mediated effect could be independent of TGF-β signaling, as AngII appears capable of activating the Smad pathway independent of TGF-β signaling (86). In addition, administration of a TGF-β neutralizing antibody in AngII-infused normocholesterolemic mice disrupts their resistance to aneurysm formation, implying a seemingly controversial protective effect of TGF-β instead (87). Further, increased AngII-mediated and insulin-like growth factor 1–mediated signaling, independent of TGF-β signaling, is thought to drive a form of inherited nonsyndromic thoracic aortic aneurysms associated with missense mutations in the *MYH11* gene (57). Interestingly, our proteomic data revealed a 7-fold decrease in the protein levels of MYH11 in the SMAs of smLRP1^–/–^ mice. Given the complexity of the multiple interactions of the AngII signaling pathway and the expansiveness of our proteomic analysis, additional studies are warranted to determine the role of LRP1 in these other signaling pathways and elucidate potential signaling crosstalk of LRP1 with the renin-angiotensin pathway.

In summary, our studies have demonstrated a critical role for LRP1 in maintaining an appropriate vSMC phenotype and in attenuating excessive AngII-mediated signaling events in the SMA. Given that little is known about mechanisms associated with splanchnic artery aneurysms in humans, our studies raise the possibility that LRP1 may play a critical role in regulating the integrity of this vasculature in humans as well, and it will be important in future studies to determine if LRP1 missense mutations are associated with splanchnic artery aneurysms.

## Methods

### Animals.

All mice were weaned at 3–4 weeks of age, maintained on a 12-hour light/12-hour dark cycle, fed a standard laboratory rodent diet (4% wt/wt fat; Envigo 2018SX), and given standard drinking water ad libitum. Mice that received drugs were provided losartan (0.6 g/L) dissolved in drinking water or ASO via subcutaneous injections. Embryonic deletion of *Lrp1* in vSMCs was achieved by crossing transgenic mice expressing Cre recombinase under the control of an SM22 SMC-specific promoter with mice expressing *loxP* sites flanking the *Lrp1* gene (provided by J Herz, University of Texas Southwestern Medical Center, Dallas, Texas, USA). The resulting offspring, *Lrp1^fl/fl^* *SM22-Cre^–/–^* (LRP1^+/+^) and *Lrp1^fl/fl^* *SM22-Cre^+/–^* (smLRP1^–/–^), were used in experimental studies with LRP1^+/+^ littermates serving as controls.

### Ultrasonography.

SMAs were scanned using a Vevo 3100 ultrasound system with an MS550 transducer (FUJIFILM VisualSonics Inc.). Mice were placed on a heated platform (37°C) to avoid hypothermia and anesthetized with isoflurane (1–2% vol/vol) to adjust the heart rate between 400 and 550 beats/minute. Color Doppler was used to confirm the pulsatile flow of the abdominal aorta. Then the probe was moved from the diaphragm caudally to visualize the SMA. A cine loop of the SMA was captured to define the maximum dilation of the SMA. Maximal luminal diameters were measured on the captured images using Vevo LAB 3.1.1 software (FUJIFILM VisualSonics Inc.).

### Microfil injection.

Mice were euthanized by an overdose of ketamine and xylazine cocktail (90 and 10 mg/kg, respectively). The thoracic cavity was cut open, and the right atrium was nicked to allow the exit of blood flow. Saline (10 mL) was perfused through the left ventricle using a pressure-controlled peristatic pump (PS/200, Living Systems Instrumentation) at physiological pressure. Directly after perfusion, the right atrium was sealed, and Microfil (Flow Tech, Inc.) was injected through the same catheter at physiological pressure. Once Microfil was visualized in the arterioles surrounding the small intestine, the pump was stopped, the catheter was clamped shut to prevent backflow of Microfil into the thoracic cavity, and the animal was set aside to allow the compound to harden (~90 minutes).

### Micro-CT scanning and 3D reconstruction.

After Microfil perfusion, animals were scanned using a Skyscan 1276 micro-CT (Bruker), and images were acquired with a pixel size of 20 μm at 2,016 × 1,344 resolution. CT scans were reconstructed using the NRecon program (Bruker) to adjust for beam hardening and ring artifacts. Image sets were saved as DICOM or BMP files (~1,200–1,500 images/animal). Reconstruction in 3D was performed using the 3D Slicer program. All bone and vasculatures not of interest were removed using the scissors tool within the program to display the aorta and its major branches. To visualize SMAs, all the other vasculatures were removed using the scissors tool.

### AGT ASO experiments.

AGT ASOs were provided by Ionis Pharmaceuticals. PBS alone (control) or AGT ASO (80 mg/kg) was injected subcutaneously on days 1 and 4 in male LRP1^+/+^ and smLRP1^–/–^ littermates when they were 6 weeks of age. Subsequently, either PBS or AGT ASO (40 mg/kg) was injected once every week for 11 weeks. At termination (18 weeks of age), plasma was collected to measure AGT concentrations.

### Plasma AGT and renin measurements.

Plasma AGT concentrations were measured using a mouse AGT ELISA kit (ab245718; Abcam). Plasma renin concentrations were measured using an ELISA kit (IB59131, Immuno-Biological Laboratories Co., Ltd.) in which the angiotensin I product was determined after incubation of plasma with recombinant mouse AGT at 37°C for 1 hour.

### SMA tissue collection.

SMA tissue was collected for proteomic and histological analysis. Mice that were designated for proteomic quantification were euthanized by CO_2_ asphyxiation, and the SMAs were collected. The adventitia and periadventitial fat from the SMAs were removed in cold PBS; then the SMA was immediately snap-frozen and stored at –80°C until analysis. For histological analysis, SMAs were collected after micro-CT images were acquired. Residual Microfil contrast reagent was removed from the SMA. The tissue was then fixed again in 4% paraformaldehyde overnight, then placed in 70% ethanol solution in preparation for decalcification, sectioning, and staining. Tissue cross sections of 5 μm thickness were sliced and stained by H&E or EVG. Morphometric measurements were performed using EVOS FL Auto Imaging System software (Invitrogen, Thermo Fisher Scientific) and ImageJ (NIH). All measurements were performed while blinded to the sample identification.

### Carotid artery ligation.

Ligation of the left common carotid artery was performed on male LRP1^+/+^ and smLRP1^–/–^ mice at 12–16 weeks of age. Mice were placed in an induction chamber and anesthetized with 3% vaporized isoflurane (Fluriso; VetOne 502017) in oxygen flowing at 1 L/min. Sedated mice were laid supine on a heating pad and maintained on 2.5% vaporized isoflurane in oxygen via nose cone. The neck area was administered a depilatory (Nair) to remove hair, disinfected with alternating 7.5% povidone-iodine (Betadine Surgical Scrub; Purdue Pharma NDC 67618-151-16) and 70% isopropyl alcohol (Webcol Alcohol Preps; Covidien 5033), and an incision was made from the sternum to the area just below the chin. The underlying fascia and glandular tissues were separated, and the exposed muscle layer was dissected carefully and retracted. The left common carotid artery was separated from the surrounding fascia and adjacent vagus nerve, and the isolated vessel was permanently ligated proximal to the carotid bifurcation using a sterilized 4-0 silk suture to fully obstruct blood flow. The incision was sutured using a 4-0 PDO absorbable monofilament suture (AD Surgical M-D430T17), and animals were weighed and administered 0.05 mg/kg buprenorphine hydrochloride (Buprenex; Reckitt Benckiser NDC 12496-0757-5) diluted in 0.9% sodium chloride injection, USP (Hospira NDC 0409-4888-02), via subcutaneous injection before returning to a cage placed on a heating pad. Animals were monitored for recovery from anesthesia and ambulatory movements. Two additional injections of 0.05 mg/kg Buprenex were administered within 24 hours of surgery at ≥6-hour intervals.

Immediately following surgery, animals were given water ad libitum supplemented with or without losartan potassium (0.6 g/L; Aurobindo Pharma Limited NDC 65862-201-99) for 4 weeks. During administration, animals were monitored for changes in appearance, activity, and food and water intake, and body weights were recorded twice per week. Four weeks postsurgery, animals were euthanized by CO_2_ asphyxiation, the right common carotid artery and ligated left common carotid artery were dissected, and the adventitia was removed from each tissue. All tissues were frozen immediately on dry ice and stored at ≤–70°C for protein analyses. For histological analysis, the whole neck and head were prepared as described below.

### Blood pressure measurements.

Blood pressure measurements in mice were obtained using the CODA High Throughput Non-Invasive Blood Pressure System (Kent Scientific Corporation CODA-HT4). Blood pressure measurements were recorded in LRP1^+/+^ and smLRP1^–/–^ mice after weaning at 3–4 weeks of age per the protocol detailed in Daugherty et al. (88). Noninvasive measurements of systolic and diastolic blood pressures were averaged over approximately 15 recorded cycles. Measurements were repeated if the standard deviation was greater than 30 mmHg. Blood pressures were taken daily for 2 weeks to allow mice to acclimate to the device. The remaining measurements were taken 3 times each week for the remaining length of the experiment.

### Carotid artery histology and vessel morphometry.

The whole neck and head were dissected from LRP1^+/+^ and smLRP1^–/–^ mice subjected to left carotid artery ligation with and without losartan. Samples were then skinned and fixed in 10% buffered formalin phosphate (fixative solution; Thermo Fisher Scientific SF100-20) for 3 days, with fixative solution exchanged for fresh fixative solution once per day. After 3 days of fixation, samples were placed in 70% ethanol solution and transferred to the Center for Vascular and Inflammatory Diseases Histology Core at the University of Maryland School of Maryland or shipped to Histoserv, Inc. for decalcification, sectioning, and staining. Whole-neck serial cross sections of 5 μm thickness were sliced starting from the carotid bifurcation to the area inferior to the lesion apex. The apex of the lesion area was identified by analyzing serial sections at 100 μm intervals by H&E, EVG, and Masson’s trichrome staining. Morphometric measurements were performed using EVOS FL Auto Imaging System software (Invitrogen, Thermo Fisher Scientific). All measurements were performed while blinded to the sample identification.

### Global quantification of protein expression.

SMA tissue from 14-week-old WT and smLRP1^–/–^ mice was rinsed in PBS to remove blood, frozen with liquid nitrogen in a tissueTUBE TT05M (Covaris catalog 520071), and impact pulverized with a cryoPREP CP01 (Covaris catalog 500230). Fractured tissue was transferred to a 1 mL milliTUBE containing an AFA fiber (Covaris catalog 520135) in 200 μL of 50 mM HEPES pH 8.5, 150 mM NaCl, and 2% Triton X-114 and sonicated with an M220 Focused-Ultrasonicator (Covaris catalog 500295). Sonication parameters were temperature 15°C, peak power 75 W, duty factor 26, cycles/burst = 1,000, and duration 600 seconds. Extracted proteins were clarified of insoluble material by centrifugation at 15,000*g* for 20 minutes at 4°C. Protein concentrations were determined with the Micro BCA colorimetric assay (Pierce, Thermo Fisher Scientific) with the addition of SDS to a final concentration of 1% in the assay solvent to prevent detergent clouding.

Aliquots containing approximately 5 μg of protein were processed using the SP3 protocol as described (89) with some modifications. Briefly, the sample aliquots were brought to 50 μL volume, and disulfide bonds were reduced and alkylated simultaneously with 10 mM TCEP, 40 mM 2-chloroacetamide in 50 mM HEPES pH 8.5, and 1% sodium deoxycholate at 70°C for 10 minutes, then cooled on ice. Proteins were precipitated and captured following addition of 10 μL of a washed 10 μg/μL suspension of SpeedBeads (Cytiva) and 400 μL of ethanol. After shaking for 10 minutes at room temperature, the beads were magnetically captured and washed 3 times with 200 μL of 80% ethanol in water. Proteins were digested on the beads in 50 μL of 50 mM HEPES pH 8.5, 1% sodium deoxycholate, and 10 ng/μL trypsin (Promega) overnight at room temperature with shaking sufficient to maintain the beads in suspension. The digest was diluted 10-fold with 80% acetonitrile and 1% formic acid, then separated from the beads magnetically, and the resulting peptides were captured on 2 mm discs of Empore Cation (CDS Analytical) fitted into 1,000 μL pipette tips (Sartorious catalog 791000). Detergents and other contaminants were removed by washing the tips serially with 1) ethyl acetate; 2) 80% acetonitrile, 1% formic acid; and 3) 10% acetonitrile, 0.2% formic acid. Peptides were eluted directly into injection vials with freshly prepared 80% acetonitrile and 5% ammonium hydroxide and immediately dried down in a centrifugal vacuum evaporator.

One-fifth of the recovered peptides from each sample was subsequently analyzed by liquid chromatography-tandem mass spectrometry. In-house capillary columns were constructed from 360 μm OD and 100 μm internal diameter × 30 cm fused silica tubing (Molex) with laser-pulled tips (Sutter Instruments) and were packed with Reprosil-PUR 3 μm C18-AQ (Dr. Maisch GmBH). Solvents A and B consisted of 0.1% formic acid in water and 80% acetonitrile with 0.1% formic acid, respectively. A 180-minute linear gradient from 2% to 35% solvent B was used for chromatographic separation. Peptides were analyzed with an Orbitrap Elite (Thermo Fisher Scientific) mass spectrometer using nano-electrospray ionization with an applied voltage of 1,800 V. MS1 spectra were acquired at a resolution of 120,000, and the 15 most abundant precursor ions were selected for fragmentation by higher energy collision dissociation. MS2 spectra were acquired at a resolution of 15,000. Dynamic exclusion parameters were list size of 500, mass window of ±7 ppm, and duration of 1 minute. Automatic gain control settings were MS1 target 1 × 10^6^, maximum inject time 100 ms; MS2 target 4 × 10^4^, maximum inject time 100 ms.

### Mass spectrometry data analysis.

Spectrum matching and protein identification and validation were performed with MSFragger (90), and quantification of protein intensities with matching between runs was performed with IonQuant (91) as components of the FragPipe analysis pipeline using the default settings of each module. The protein database used for the search was the *Mus musculus* reviewed sequence database downloaded from UniProt on March 8, 2022. The results were subsequently processed to filter out common contaminants, decoy hits from the reverse database, and protein groups identified by a single peptide. The data were filtered as follows: (a) binary expression of a protein (i.e., protein exclusively identified in either LRP1^+/+^ or smLRP1^–/–^) was only considered relevant if all LRP1^+/+^ samples or all smLRP1^–/–^ samples expressed the protein. The missing values were imputed with the minimum intensity value for each specific data set; (b) for samples expressed in both LRP1^+/+^ and smLRP1^–/–^ tissue, the filtering process required 2 or more proteins to be detected in both the LRP1^+/+^ and smLRP1^–/–^ samples. False discovery analysis was performed using the Benjamini, Krieger, and Yekutieli method (92) using GraphPad Prism 9.0 software. Causal analysis of proteomic data was performed (52) in IPA upstream analysis software (QIAGEN). For IPA, the binary values were imputed using local minimum intensities. Enrichment analyses for gene ontology (biological process) were performed using clusterProfiler 4.2.2 R package on R 4.1.0. The mass spectrometry proteomics data have been deposited to the ProteomeXchange Consortium via the PRIDE (93) partner repository with the data set identifier PXD038236.

### Statistics.

Prism 9.0 (GraphPad Software) was used for statistical analysis. Normality was determined on data sets using the Shapiro-Wilk test. To compare variables between 2 groups, an unpaired 2-tailed Student’s *t* test was used for normally distributed variables. When the effect of 2 variables was analyzed in data sets containing normally distributed variables, a 2-way ANOVA with Tukey’s post hoc test was used. To compare more than 2 groups in which normality was not met, the variables were analyzed by a 1-way ANOVA on ranks (Kruskal-Wallis nonparametric test with Dunn’s multiple-comparison post hoc test). All results are presented as mean ± SEM, with *P* values shown above bars. A *P* ≤ 0.05 was set as the threshold for significance.

### Study approval.

All animal studies were approved by the Institutional Animal Care and Use Committee of the University of Maryland School of Medicine or the University of Kentucky.

## Author contributions

JZ, DTA, MF, BOA, BH, MM, FN, AEM, MMW, MF, JJM, DAH, and PW conducted experiments and acquired and analyzed data. JZ, DTA, HS, BH, MM, FN, AAU, HSL, SCM, AD, HS, HSL, AD, and DKS analyzed data. JZ, DTA, HSL, HS, AD, and DKS wrote and edited the manuscript.

## Supplementary Material

Supplemental data

## Version 1 12/06/2022

In-Press Preview

## Version 2 01/24/2023

Electronic publication
