# Supplementary material for: LRP1 protects against excessive superior mesenteric artery remodeling by modulating angiotensin II–mediated signaling
Source: JCI Insight. 2023 Jan 24;8(2):e164751. doi: 10.1172/jci.insight.164751 (PMC9977308; doi:10.1172/jci.insight.164751)
Supplement: Supplemental data [file jciinsight-8-164751-s124.pdf]

**Supplemental data**

# LRP1 protects against excessive superior mesenteric artery remodeling by modulating angiotensin II-mediated signaling

**Table I. Fold change in integrins and associated proteins in smLRP1-/- mice**

| Protein ID | Log2 FC KO/LRP1+/+ | FDR value |
|---|---|---|
| ITGB2 | 1.60 | 0.0013 |
| ITGAM | 0.82 | 0.0301 |
| ITGAV | -0.38 | 0.0403 |
| ITGA1 | -0.69 | 0.0338 |
| TLN1 | -0.71 | 0.0009 |
| ITGB1 | -0.94 | 0.0019 |
| ITGA5 | -0.96 | 0.0056 |
| ITGA3 | -0.99 | 0.0036 |
| ITGA9 | -0.99 | 0.0025 |
| ITGA7 | -1.07 | 0.0010 |
| FERMT2 | -1.20 | 0.0012 |
| ITGB3 | -1.26 | 0.0002 |
| ITGA8 | -1.71 | 0.0020 |

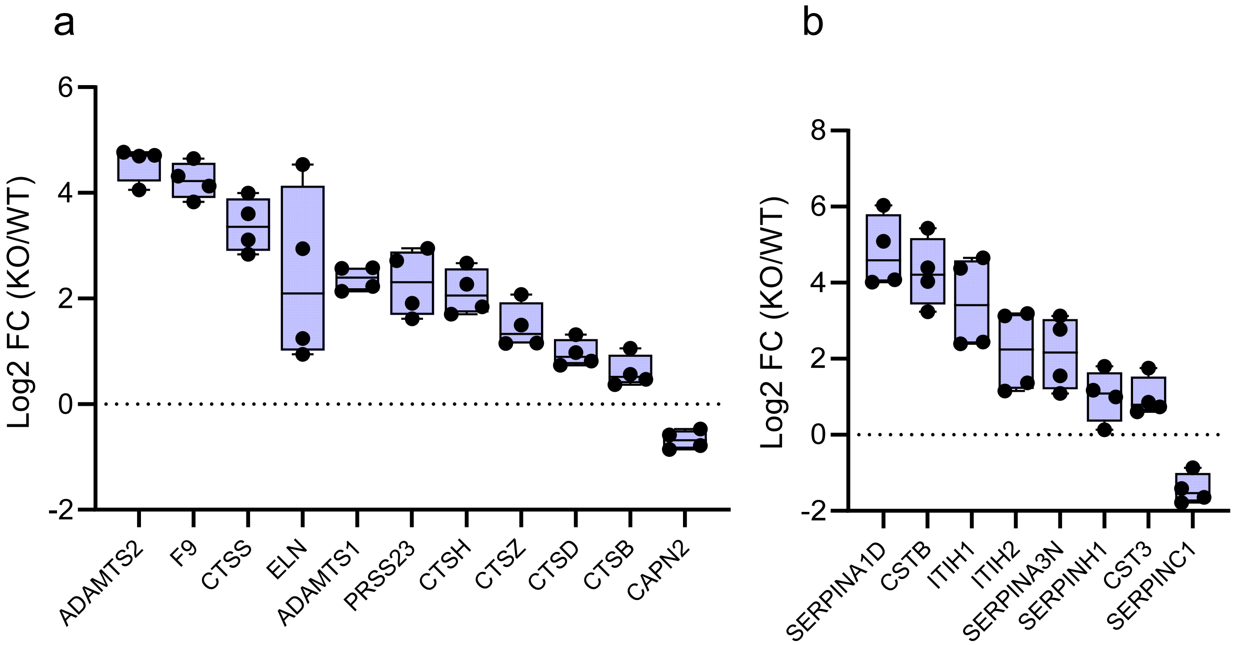


**Supplemental Figure 1. Proteinases (a) and proteinase inhibitors (b) are deregulated in smLRP1-/- mice as revealed by mass spectrometry.** Log2 fold change in several proteinases (a) or proteinase inhibitors (b) whose levels are altered in the SMA of smLRP1-/- as detected by mass spectrometry.

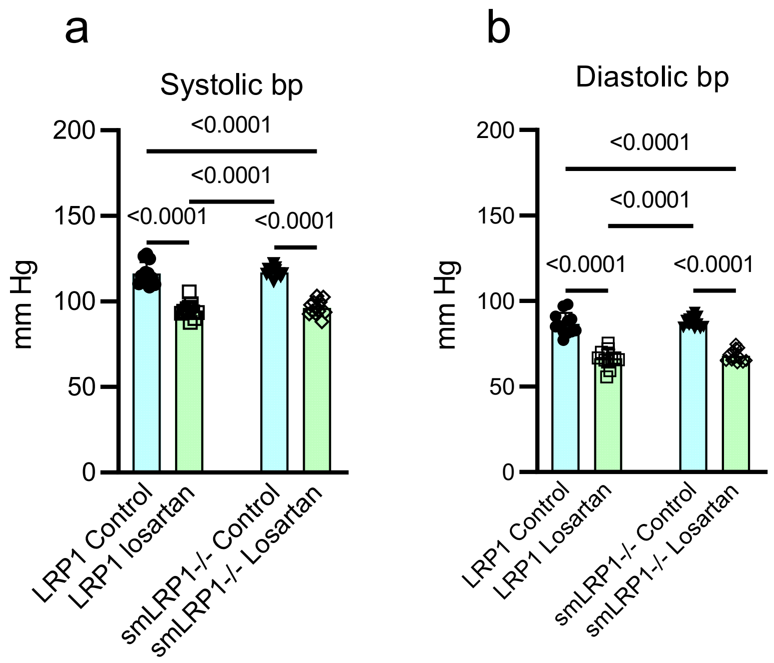


**Supplemental Figure 2. Losartan administration reduced blood pressure in both LRP1+/+ and smLRP1-/- mice.** After weaning at 3-4 weeks of age, mice were provided with or without losartan (0.6 g/L drinking water) and kept on the drug for 12 weeks before analysis. For blood pressure measurements, non-invasive measurements of systolic and diastolic blood pressures were averaged over approximately 15 recorded cycles and were taken daily for two weeks to allow mice to acclimate to the device. The remaining measurements were taken three times each week for the remaining length of the experiment. One way ANOVA with Dunnett's multiple comparison test

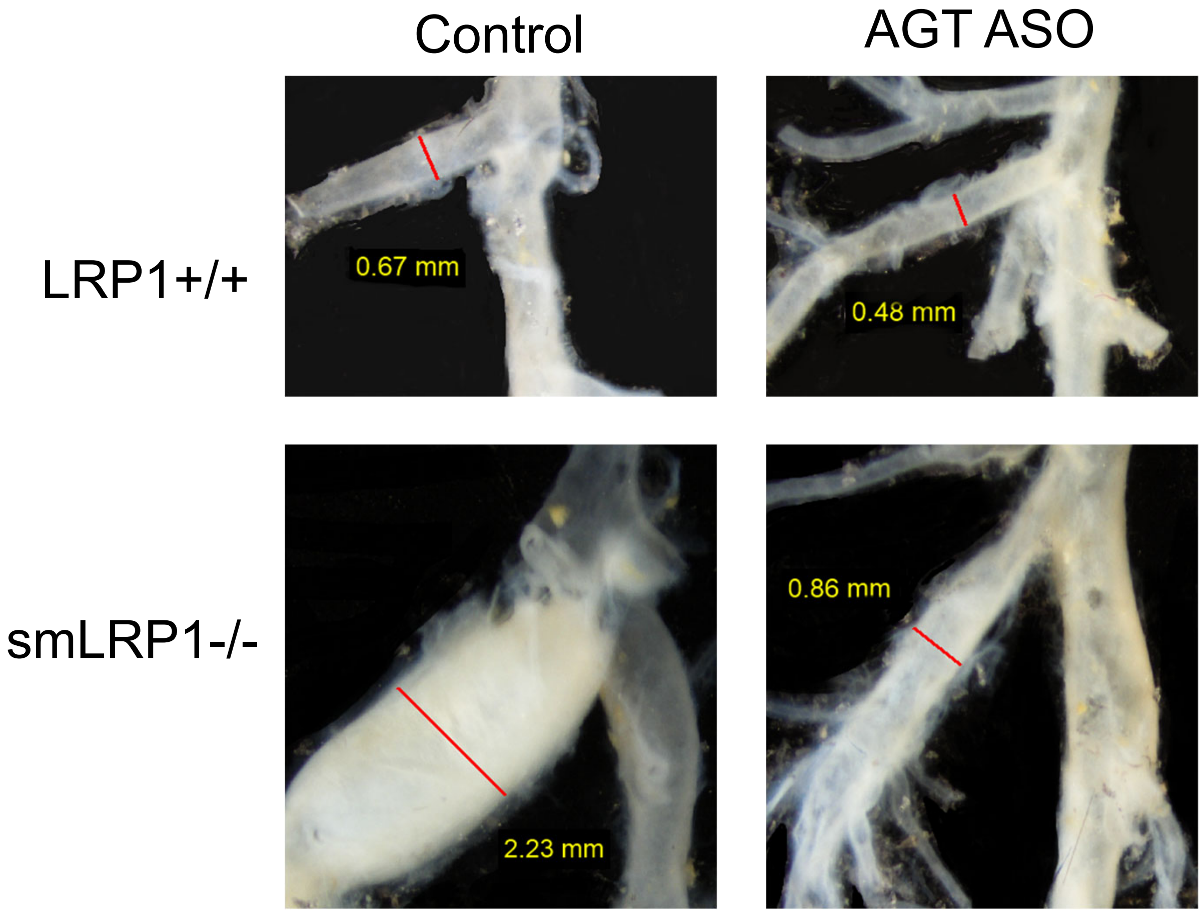


**Supplemental Figure 3. Ex-vivo measurement of SMA diameter for LRP1+/+ and smLRP1-/- mice receiving AGT ASO**

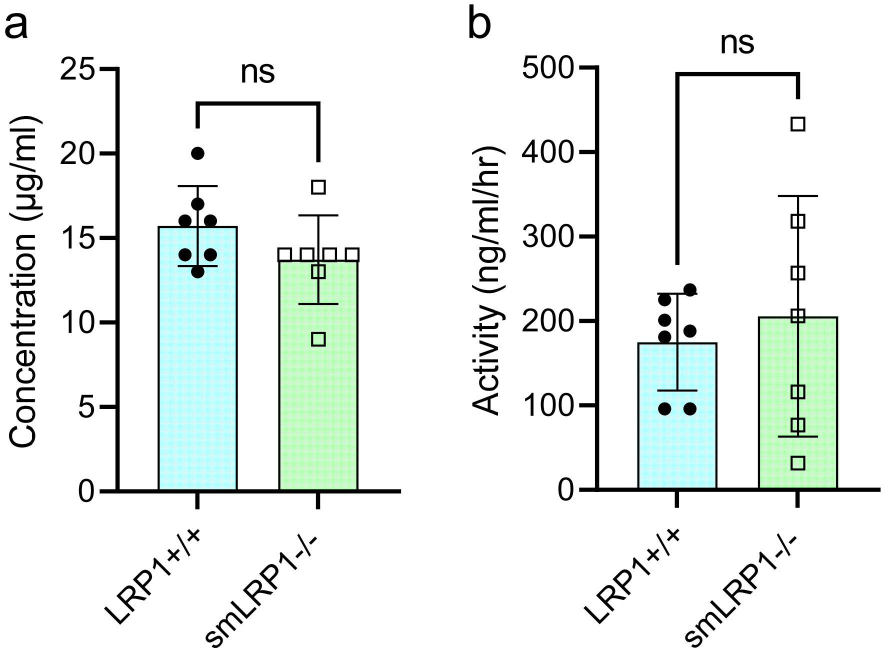


**Supplemental Figure 4. Angiotensinogen and renin concentrations in plasma of LRP1+/+ and smLRP1-/- mice are similar**. (a) Plasma collected from 16–18 week old LRP1+/+ and smLRP1-/- mice were assayed for AGT concentrations by ELISA. (b) Renin activity concentrations were measured by incubating plasma with recombinant mouse AGT at 37°C for 1 hour, and then quantifying the amount of angiotensin I product formed. (Students *t* test)
